# *In vitro* characterization of coconut waste–derived indigenous microorganisms as probiotic and synbiotic candidates for sustainable poultry production

**DOI:** 10.14202/vetworld.2026.745-759

**Published:** 2026-02-26

**Authors:** Hera Dwi Triani, Muhammad Amri, Toni Malvin, Ibran Eka Putra, Wulansih Dwi Astuti, Gusri Yanti, Resolinda Harly, Yetti Marlida, Roni Pazla

**Affiliations:** 1 Department of Agricultural Extension, Faculty of Science, Social and Education, Universitas Prima Nusantara Bukittinggi, 26122, Indonesia; 2 Department of Animal Husbandry and Animal Health, Politeknik Pertanian Negeri Payakumbuh, 26271, Indonesia; 3 Department of Veterinary Medicine, Faculty of Veterinary Medicine, Universitas Negeri Padang, 26136, Indonesia; 4 Research Center for Applied Zoology, National Research and Innovation Agency, Cibinong, West Java, 16911, Indonesia; 5 Department of Nutrition and Feed Technology, Faculty of Animal Science, Universitas Andalas, Padang, 25163, Indonesia

**Keywords:** acid tolerance, antimicrobial activity, coconut-waste, *Escherichia coli*, lactic acid bacteria, poultry probiotics, short-chain fatty acids, synbiotic feed additive

## Abstract

**Background and Aim:**

The global restriction on antibiotic growth promoters (AGPs) in poultry production due to antimicrobial resistance concerns has accelerated the search for effective, sustainable alternatives. Probiotics derived from agricultural by-products offer a promising strategy to enhance gut health and productivity while reducing environmental waste. Coconut-waste, including coconut water and pulp, is rich in fermentable substrates that support the growth of lactic acid bacteria (LAB) and the production of functional metabolites. This study aimed to perform an integrated *in vitro* characterization of indigenous microorganisms derived from coconut-waste fermentation as potential probiotic and synbiotic candidates for sustainable poultry production.

**Materials and Methods:**

Indigenous microorganism solutions (IMOS) were produced through anaerobic fermentation of coconut water and coconut pulp for 5, 10, 15, and 20 days using a completely randomized design with four treatments and five replicates. Physicochemical properties (pH, LAB counts), enzymatic activities (cellulase and mannanase), tolerance to simulated gastrointestinal conditions (acidic pH 2.5, bile salts at 0.3% and 0.5%, and thermal exposure at 42°C), cell surface hydrophobicity, antimicrobial activity against *Escherichia coli*, *Salmonella* spp., and *Staphylococcus aureus*, and short-chain fatty acid (SCFA) production were evaluated using standard microbiological and analytical methods.

**Results:**

Fermentation duration significantly influenced all evaluated parameters (p < 0.05). IMOS fermented for 15 days exhibited the lowest pH (3.19 ± 0.02), the highest LAB population (2.05 ± 0.13 × 10¹¹ CFU/mL), optimal cellulase (12.50 ± 0.15 U/mL) and mannanase activities (20.48 ± 0.13 U/mL), and the greatest cell surface hydrophobicity (95.09 ± 0.35%). LAB survival remained high under simulated gastrointestinal stress, reaching 80.23 ± 4.12% at pH 2.5 (6 h), 71.45 ± 0.56% in 0.5% bile salts, and 8.09 ± 0.35 × 10¹¹ CFU/mL at 42°C. Antimicrobial assays demonstrated complete inhibition of *E. coli* after 24 h at 15 days of fermentation. Acetate (3.34–3.43 g/L) and butyrate (0.66–0.71 g/L) were the dominant SCFAs detected.

**Conclusion:**

Coconut waste–derived IMOS demonstrates strong in vitro probiotic and synbiotic characteristics and represents a low-cost, environmentally sustainable functional feed additive for poultry. Fermentation for 15 days yielded optimal functional properties. Further in vivo validation is warranted to confirm efficacy under practical production conditions.

## INTRODUCTION

The application of probiotics as a natural alternative to antibiotics and growth promoters (AGPs) for improving poultry productivity has received increasing attention. Growing concerns regarding antibiotic resistance, environmental impacts [1], and adverse effects on human health [2, 3] have positioned probiotics as a sustainable strategy for producing safe and high-quality poultry products, including meat and eggs.

As probiotic candidates, lactic acid bacteria (LAB) contribute to strengthening the intestinal mucosal barrier, suppressing pathogenic microorganisms, and synthesizing bioactive metabolites, such as short-chain fatty acid (SCFA) that support immune function and gut health [4–5]. Previous studies have demonstrated that LAB derived from fermentation residues possess strong probiotic potential, exhibiting tolerance to acidic and bile salt conditions as well as antimicrobial activity against a range of pathogens [6–7]. Dietary supplementation with LAB-based probiotics has been shown to enhance poultry growth performance and improve meat quality [8, 9].

Indigenous microorganism solutions (IMOS) represents a promising, low-cost, and locally available probiotic source. Coconut by-products provide fiber- and nutrient-rich substrates that favor the growth and activity of probiotic microorganisms. Coconut water, pulp, and residues supply fermentable carbon sources, oligosaccha-rides/mannan, minerals, and nitrogen that support LAB proliferation and metabolic activity during fermentation. Mannanase activity within IMOS facilitates the release of mannan-oligosaccharides, which function as prebiotics, while SCFA production contributes to pathogen suppression and overall gut health [10, 11]. A previous study reported that IMOS-derived from fermented coconut-waste, composed of coconut water and coconut pulp, achieved LAB populations of up to 5.4 × 10¹¹ CFU/mL, with a pH of 3.3 and substantial cellulase and mannanase activities [12].

Despite growing interest in probiotics derived from fermented agricultural by-products, comprehensive evaluations of IMOS as probiotic candidates remain limited. Existing studies on coconut-based fermentations have largely focused on isolated attributes, such as microbial enumeration, organic acid production, or enzyme activity, without integrating multiple functional criteria within a single system. To date, no study has simultaneously assessed LAB viability, tolerance to simulated gastrointestinal conditions (acidic pH, bile salts, and elevated temperature), antimicrobial activity against poultry-relevant pathogens, enzymatic activities related to fiber and mannan degradation, and SCFA production in a coconut waste–based IMOS matrix. This lack of integrated in vitro characterization constrains a holistic understanding of the functional probiotic and synbiotic potential of IMOS-derived from coconut-waste and limits its rational development as an alternative to AGPs in poultry production.

Therefore, this study aimed to evaluate IMOS as a potential natural probiotic candidate for poultry by comprehensively assessing LAB counts, tolerance to simulated gastrointestinal conditions (acid, bile salts, and temperature), pathogen inhibition capacity, and SCFA production. IMOS is of particular interest due to its local availability, cost-effectiveness, prebiotic content in the form of MOS, and enzymatic activities that support probiotic functionality. This study specifically addresses challenges associated with the AGP ban and the high pathogen burden in poultry production systems. We hypothesized that indigenous microorganisms derived from coconut-waste fermentation would exhibit high LAB viability, strong tolerance to gastrointestinal stressors, effective antimicrobial activity against poultry pathogens, and the capacity to produce beneficial SCFAs. By integrating microbial viability, enzymatic functionality, antimicrobial properties, and SCFA production within a single fermentation system, this work provides a comprehensive *in vitro* characterization and highlights the potential of coconut water and pulp–based IMOS as a sustainable probiotic source for poultry production.

## MATERIALS AND METHODS

### Ethical approval

This study did not involve live animals, human participants, or vertebrate tissues. All analyses were conducted *in vitro* using microorganisms derived from fermented coconut-waste and standard bacterial strains. Therefore, ethical approval was not required.

### Study period and location

The study was conducted between April and July 2025 at the Veterinary Laboratory, Faculty of Veterinary Medicine, Universitas Negeri Padang, and the Biotechnology Laboratory, Faculty of Animal Science, Universitas Andalas, Indonesia.

### Materials

#### Raw materials

Fresh coconut water and coconut pulp were obtained from a local supplier in Bukittinggi, Indonesia. The coconuts used were of medium maturity (7–8 months).

#### Chemicals and reagents

All chemicals and reagents used in this study were of analytical grade and obtained from standard commercial suppliers.

#### Microbiological media

De Man, Rogosa and Sharpe (MRS) broth and MRS agar were used for LAB cultivation and enumeration. Eosin methylene blue agar and mannitol salt agar were used for the isolation and enumeration of *E. coli* and *S. aureus*, respectively.

#### Reference bacterial strains

LAB were derived from the IMOS fermentation system. Pathogenic test bacteria, including *E. coli*, *Salmonella* spp., and *S. aureus*, were obtained from the microbiology laboratory culture collection and used as reference strains for antibacterial assays.

### Experimental design

IMOS was produced from coconut water and coconut pulp at a ratio of 1 L coconut water to 500 g coconut pulp and subjected to anaerobic fermentation for 5 (T1), 10 (T2), 15 (T3), and 20 days (T4). Coconut water and coconut pulp were obtained from a single local supplier in Bukittinggi, Indonesia, using coconuts of medium maturity (7–8 months). The coconut pulp was washed with sterile distilled water, manually crushed under hygienic conditions, and processed within 2 h of collection to prevent microbial degradation.

Fermentation was conducted using a completely randomized design with four treatments and five biological replicates per treatment. Anaerobic fermentation was performed at ambient laboratory temperature (28°C–30°C) in tightly sealed, food-grade plastic containers (5 L capacity). Fermentation pH was measured at the beginning and end of each fermentation period (days 5, 10, 15, and 20).

**Figure 1:**
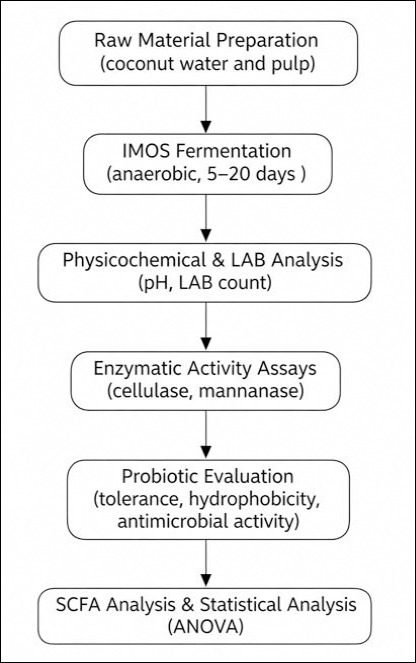
Experimental workflow for the production and evaluation of an indigenous microorganism solution.

The resulting IMOS was evaluated for physicochemical characteristics (pH and total plate count [TPC]), enzymatic activities (cellulase and mannanase), LAB tolerance to gastrointestinal conditions (acid, bile salt, and thermal tolerance), cell surface hydrophobicity, antimicrobial activity against pathogenic bacteria, and short-chain fatty acid (SCFA) production. Enzyme assays and physicochemical analyses were conducted in triplicate, whereas antimicrobial assays and SCFA analyses were performed using triplicate samples per treatment. Quality control measures included aseptic handling throughout all procedures, the inclusion of appropriate blanks and controls, and acceptance of calibration curves only when R² ≥ 0.99. Figure 1 illustrates the experimental workflow.

### LAB colonies and pH

The TPC of IMOS was assessed using the serial dilution technique with 0.85% peptone solution. From each dilution, 0.1 mL was inoculated onto MRS agar plates and incubated at 37°C for 24 h. The resulting colonies were quantified as CFU/mL and transformed to log_10_ values.

Before each assay, LAB cultures were grown in MRS broth at 37°C for 24 h. Cell density was adjusted to approximately 10^8^ CFU/mL using a 0.5 McFarland standard. Freshly prepared standardized LAB suspensions were used immediately for subsequent assays. When short-term storage was required, cultures were maintained at 4°C for no longer than 24 h to minimize viability loss.

pH was measured using a digital pH meter calibrated with standard buffer solutions (pH 4.01 and 7.00). The electrode was immersed directly into homogenized IMOS samples at room temperature until stable readings were obtained.

### Cellulase activity

Cellulase activity was determined using the Nelson method as modified by Jennifer *et al*. [13]. One milliliter of crude enzyme extract was mixed with 1 mL of substrate (0.5% carboxymethyl cellulose in buffer) and incubated at 40°C for 30 min in an agitated water bath. After incubation, 1 mL of Nelson’s A–B reagent was added, and the mixture was boiled for 20 min, cooled, and supplemented with 1 mL of phosphomolybdate reagent and 7 mL of distilled water. Absorbance was measured at 575 nm using a spectrophotometer, and glucose standard curves were used for calibration. One unit (U) of enzyme activity was defined as the amount of enzyme required to release 1 μmol of reducing sugar per minute.

### Mannanase activity

Mannanase activity was determined based on the amount of mannose released using a modified Nelson method [14]. One milliliter of substrate solution (0.5 g/100 mL in 50 mM phosphate buffer, pH 7) was mixed with 1 mL of enzyme solution and incubated at 50°C for 30 min. The reaction was terminated by heating at 100°C for 5 min, followed by centrifugation for 5 min. The supernatant (1 mL) was mixed with Nelson reagent, boiled for 20 min, cooled, and supplemented with phosphomolybdate reagent and distilled water. Absorbance was measured at 595 nm. One unit (U) was defined as the amount of enzyme required to release 1 μmol of mannose per minute.

### Gastric pH tolerance test

Gastric tolerance was evaluated according to Dowarah *et al*. [15]. LAB isolates were tested in MRS broth adjusted to pH 2.5 using 37% HCl and untreated MRS broth as control. Bacterial suspensions (0.5 mL; 10^9^ CFU/mL) were cultured in 5 mL of each medium at 37°C for 3 and 6 h. Bacterial growth was monitored by measuring absorbance at 600 nm. Acid tolerance was expressed as survival percentage according to Tokatli *et al*. [16].

For all gastrointestinal tolerance assays, standardized LAB suspensions (approximately 10^8^ CFU/mL) were used as the initial inoculum. Samples were collected at defined time points, and viable LAB counts were determined using the plate count method on MRS agar.

### Bile salt tolerance test

Bile salt tolerance was evaluated following Nwachukwu *et al*. [17] with modifications. Oxgall was incorporated into MRS broth at final concentrations of 0.3% and 0.5%. Bacterial suspensions (0.5 mL; 10^9^ CFU/mL) were cultured in bile-supplemented media at 37°C for 5 h. Bile-free broth served as the control, and tolerance was expressed as survival percentage.

### Hydrophobicity and adhesion

Cell surface hydrophobicity was determined using the microbial adhesion to hydrocarbons method. Standardized LAB suspensions were mixed with xylene, vortexed for 2 min, and allowed to separate. The OD_600_ of the aqueous phase was measured before and after adhesion. Hydrophobicity (%) was calculated as (1 − A1/A0) × 100.

Bacterial adhesion to a solid surface was evaluated following El-Jeni *et al*. [18]. LAB cultures were incubated with stainless-steel plates at 37°C for 24 h. Plates were rinsed, immersed in sterile 1% peptone water, and vortexed to detach adherent cells, which were enumerated on MRS agar.

Thermal tolerance was evaluated by incubating LAB suspensions at 42°C for 6 h. Viability was determined using TPC after serial dilution, and results were expressed as CFU/mL.

### Antibacterial activity

Pathogenic bacteria (*E. coli*, *Salmonella* spp., and *S. aureus*) were cultured to logarithmic phase and standardized to approximately 10^8^ CFU/mL using a 0.5 McFarland standard. IMOS was mixed with each pathogen and incubated at 37°C for 0, 24, and 36 h. Samples were plated on selective media, and inhibition was calculated as a percentage reduction relative to untreated controls.

### SCFA analysis

SCFA concentrations were analyzed by gas chromatography (GC) coupled with a flame ionization detector (GC-FID) following Zhao *et al*. [19]. Samples were centrifuged, filtered, mixed with isobutyric acid as an internal standard, and injected in split mode. Acetate, propionate, and butyrate were quantified using external calibration curves (R² ≥ 0.99) and expressed as g/L.

### Statistical analysis

Statistical analyses were performed using the Statistical Package for the Social Sciences version 26 (IMB Corp., NY, USA). Data were tested for normality and homogeneity using Shapiro–Wilk and Levene’s tests, respectively. Physicochemical characteristics, LAB counts, and gastrointestinal tolerance were analyzed using analysis of variance (ANOVA) followed by Duncan’s multiple range test (DMRT). Statistical significance was declared at p < 0.05, whereas antimicrobial activity and SCFA content were analyzed using quantitative descriptive analysis.

## RESULTS

The physicochemical characteristics and total LAB are summarized in Table 1.

**Table 1:** Physicochemical characteristics and total lactic acid bacteria.

Parameters	T1	T2	T3	T4	SEM
pH	3.38^c^ ± 0.03	3.236^b^ ± 0.02	3.21^ab^ ± 0.01	3.19^a^ ± 0.02	0.01
Total lactic acid bacteria (CFU/mL)	1.38^a^ ± 0.02 × 10¹¹	1.82^b^ ± 0.06 × 10¹¹	2.05^c^ ± 0.13 × 10¹¹	1.37^a^ ± 0.11 × 10¹¹	0.05
Cellulase activity (U/mL)	10.23^a^ ± 0.23	13.91^d^ ± 0.15	12.50^c^ ± 0.15	10.83^b^ ± 0.22	0.08
Mannanase activity (U/mL)	15.08^b^ ± 0.19	20.19^a^ ± 0.55	20.48^a^ ± 0.13	15.51^b^ ± 0.33	0.16

Values represent mean ± SEM. Different superscripts within the same row indicate significant differences (p ≤ 0.05). T1 = IMOS fermented for 5 days, T2 = IMOS fermented for 10 days, T3 = IMOS fermented for 15 days, T4 = IMOS fermented for 20 days, IMOS = Indigenous microorganism solution, SEM = Standard error of the mean.

### pH value

**Figure 2:**
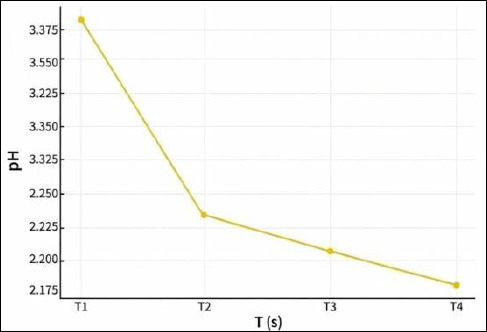
Changes in pH values of the indigenous microorganism solution during fermentation.

Fermentation duration significantly affected the pH of the IMOS liquid (p < 0.05). Post hoc analysis using DMRT showed that the lowest pH values were recorded in treatments T3 and T4, corresponding to 15 and 20 days of fermentation, with pH values of 3.19 and 3.21, respectively. In contrast, the highest pH value was observed at the shortest fermentation period (T1, 5 days). Figure 2 illustrates the progressive decrease in pH with increasing fermentation duration.

### Total number of LAB colonies

**Figure 3:**
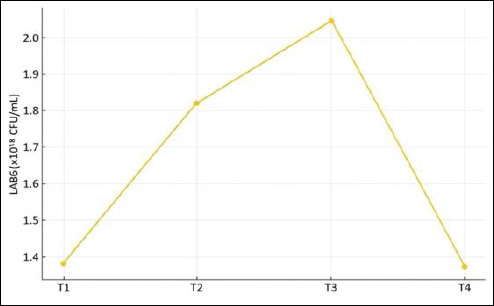
Growth pattern of lactic acid bacteria during fermentation of the indigenous microorganism solution.

LAB characteristics were evaluated at the community level, as IMOS is applied as a mixed culture rather than as individual isolates. Fermentation duration had a significant effect on total LAB counts (p < 0.01). Post hoc DMRT analysis indicated that treatment T3 exhibited the highest LAB population, which was significantly greater (p < 0.01) than those of all other treatments. Conversely, the lowest LAB counts were observed in treatments T1 and T4. The LAB growth pattern across fermentation durations is shown in Figure 3.

### Cellulase activity

Fermentation time significantly influenced cellulase activity. Further analysis revealed that treatment T2 (10 days of fermentation) exhibited the highest cellulase activity, reaching 13.91 U/mL, followed by treatment T3 (15 days of fermentation), which recorded 12.50 U/mL.

### Mannanase activity

**Figure 4:**
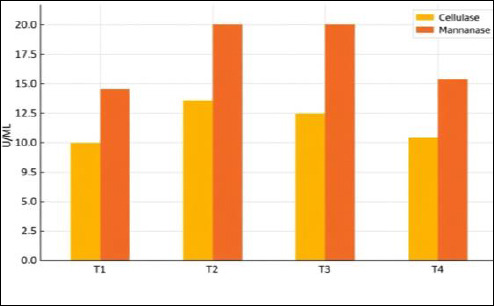
Cellulase and mannanase activities of the indigenous microorganism solution at different fermentation durations.

Fermentation duration had a highly significant effect on mannanase activity (*p* < 0.01). Post hoc DMRT analysis demonstrated that treatments T2 and T3 showed markedly higher mannanase activity than the other treatments, with values of 20.19 and 20.48 U/mL, respectively. The combined activities of cellulase and mannanase during fermentation are presented in Figure 4.

### Viability at gastrointestinal pH

LAB viability at pH 2.5 remained relatively high, ranging from 87.85% to 95.64% after 3 h of exposure and from 65.28% to 80.24% after 6 h. ANOVA indicated that fermentation duration had a highly significant effect on LAB viability at pH 2.5 after both 3 and 6 h of exposure (p < 0.01). Post hoc DMRT analysis showed that LAB viability after 3 h was significantly higher in treatment T2 (95.64%) than in all other treatments (p < 0.01). In contrast, the highest LAB viability after 6 h was observed in treatments T3 and T4, ranging from 78.46% to 80.23%.

### Bile salt tolerance

LAB derived from coconut waste–based IMOS exhibited tolerance to bile salts at concentrations of 0.3% and 0.5%. Survival rates ranged from 59.70% to 91.22% at 0.3% bile salts and from 57.05% to 71.45% at 0.5%. Fermentation duration significantly influenced bile salt tolerance. At 0.3% bile salt concentration, the highest LAB survival was observed in treatment T3 (91.22%), whereas treatment T4 showed the highest survival at 0.5% bile salts (71.45%).

### Thermotolerance under gastrointestinal conditions

**Figure 5:**
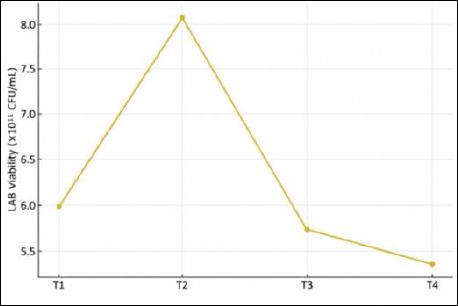
Thermal tolerance of lactic acid bacteria at 42°C during fermentation of the indigenous microorganism solution.

Fermentation duration significantly affected LAB viability at 42°C (p < 0.01). As shown in Table 2, treatment T2 (10 days of fermentation) yielded the highest LAB population at 42°C (8.09 × 10¹¹ CFU), which was significantly higher than those of the other treatments. LAB populations at 42°C were comparable between treatments T1 and T3, whereas the lowest population was observed in T4 (5.36 × 10¹¹ CFU). LAB viability at 42 °C is illustrated in Figure 5.

**Table 2:** Tolerance to gastrointestinal conditions.

Parameters	T1	T2	T3	T4	SEM
pH 2.5 (3 h, %)	93.26^bc^ ± 1.79	95.644^c^ ± 1.25	92.50^b^ ± 2.51	87.85^a^ ± 2.39	0.93
pH 2.5 (6 h, %)	75.06^b^ ± 2.51	65.29^a^ ± 2.56	78.46^bc^ ± 1.78	80.23^c^ ± 4.12	1.28
Bile salts 0.3% (%)	59.71^a^ ± 1.84	68.73^b^ ± 1.04	91.22^d^ ± 7.01	81.05^c^ ± 4.90	0.48
Bile salts 0.5% (%)	57.05^a^ ± 1.24	61.13^b^ ± 1.30	69.09^c^ ± 0.78	71.45^d^ ± 0.56	1.89
Temperature, 42 °C (CFU/mL)	5.98^a^ ± 0.64 × 10¹¹	8.09^b^ ± 0.35 × 10¹¹	5.74^a^ ± 1.25 × 10¹¹	5.36^c^ ± 0.88 × 10¹¹	0.38
Hydrophobicity (%)	93.34^c^ ± 0.21	90.09^a^ ± 0.42	95.09^d^ ± 0.35	92.24^b^ ± 0.88	0.48

Values represent mean ± SEM. Different superscripts within the same row indicate significant differences (p ≤ 0.05). T1 = IMOS fermented for 5 days, T2 = IMOS fermented for 10 days, T3 = IMOS fermented for 15 days, T4 = IMOS fermented for 20 days, IMOS = Indigenous microorganism solution, SEM = Standard error of the mean.

### Cell surface hydrophobicity

Fermentation duration significantly influenced LAB hydrophobicity (p < 0.01). Mean hydrophobicity values ranged from 90.25% to 95.09%. Treatment T3 (15 days of fermentation) exhibited a significantly higher hydrophobicity than the other treatments, reaching 95.09% (Table 2).

### Antimicrobial activity against pathogenic bacteria

**Figure 6:**
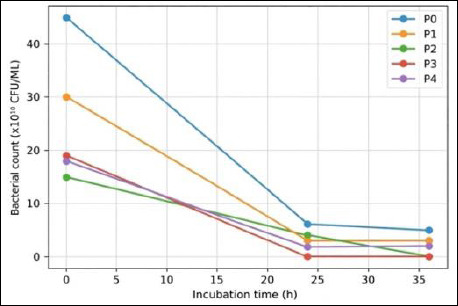
Antimicrobial activity of the indigenous microorganism solution against *Escherichia coli* at different incubation times.

**Figure 7:**
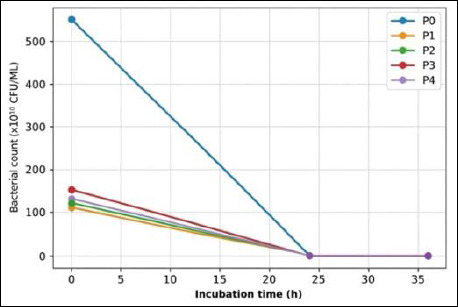
Antimicrobial activity of the indigenous microorganism solution against *Staphylococcus aureus* at different incubation times.

**Figure 8:**
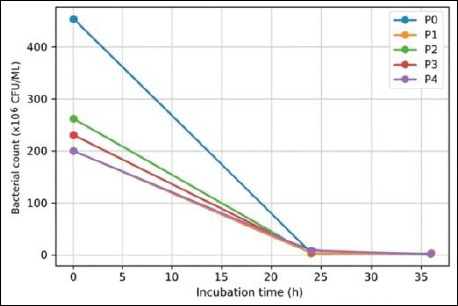
Antimicrobial activity of the indigenous microorganism solution against *Salmonella* spp. at different incubation times.

IMOS supplementation markedly suppressed the growth of pathogenic bacteria compared with the control (T0). In the absence of IMOS, pathogenic bacterial counts remained high throughout the observation period. In contrast, treatments supplemented with IMOS (P1–P4) showed a pronounced reduction in pathogenic populations, with *E. coli* being completely inhibited to undetectable levels. The antimicrobial effects of IMOS against *E. coli*, *S. aureus*, and *Salmonella* spp. are presented in Figures 6–8.

### Short-chain fatty acids

**Figure 9:**
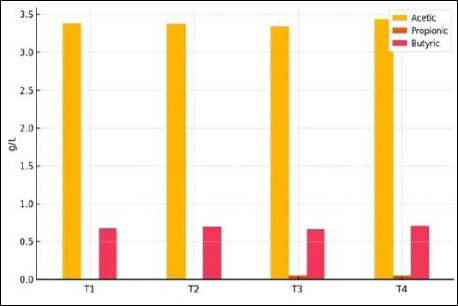
Profile of short-chain fatty acids produced during fermentation of the indigenous microorganism solution.

SCFA analysis of coconut waste–derived IMOS revealed that acetic acid was the predominant component, with concentrations ranging from 3.34% to 3.41%, followed by butyric acid (0.66%–0.71%). Only trace amounts of propionic acid were detected (0.01%–0.02%). The SCFA profile of IMOS is shown in Figure 9.

## DISCUSSION

### Physicochemical characteristics and growth of LAB

#### pH value

The lower pH observed in treatments T3 and T4 was attributed to the attainment of the exponential growth phase after 15 days of fermentation, characterized by high microbial proliferation. Consequently, the microbial population, including LAB, substantially increased, resulting in a significant reduction in pH. The pH did not decrease further when the fermentation period was extended to 20 days, indicating that the microbial community in the IMOS solution entered the stationary phase, during which no additional population growth occurred. These results indicate that the optimal fermentation condition was reached after 15 days.

In contrast, the pH value of IMOS fermented for 5 days was significantly higher than that of the other treatments. This result can be explained by the fact that the microorganisms were still in the lag or adaptation phase, during which the acid-producing microbe population had not yet reached optimal levels. The pH value is closely associated with microbial growth [20] because during fermentation, microorganisms produce metabolic compounds, particularly organic acids, which create an acidic environment and consequently lower the pH [21].

#### Total LAB

The results of this study demonstrated that LAB reached their optimal growth after 15 days of fermentation (T3), with the highest TPC recorded at 2.05 × 10¹¹ CFU/mL. At this stage, the pH was significantly low, thereby inhibiting the growth of pathogenic microorganisms. Consequently, LAB continued to proliferate as a result of reduced competition for nutrients following the decline of pathogenic microbes. LAB are Gram-positive bacteria capable of hydrolyzing carbohydrate substrates into organic acids such as propionic, acetic, and lactic acids, resulting in an inhibitory environment for the growth of spoilage microorganisms [22]. When the fermentation period was extended to 20 days, the total LAB count decreased to 1.37 × 10¹¹ CFU/mL. This decline can be attributed to the reduction of available nutrients in the IMOS medium, causing the bacterial population to enter the death phase [23].

### Enzymatic activity

#### Cellulase activity

The increased cellulase activity observed in treatment T2 is considered to result from the higher population of cellulolytic microbes that produce cellulase to degrade crude fiber. Cellulase activity is closely related to the population of cellulolytic bacteria; higher cellulase activity indicates a greater extent of fiber degradation into monosaccharides [24], which are also essential for the growth of probiotic bacteria in IMOS. In this study, the cellulase activity detected in coconut-waste–based IMOS was higher than that reported for cellulolytic bacteria isolated from cassava waste, which exhibited cellulase activities of only 6.35 and 7.58 U/mL, respectively [25]. Cellulase activity declined after reaching its optimum at T2 when the fermentation period was prolonged to 15 days (T3). This reduction is likely a result of competition between cellulolytic and mannanolytic microbes for available nutrients, causing partial mortality and a decrease in the cellulolytic bacterial population. Interestingly, mannanase activity increased in T3 as cellulase activity decreased. The simultaneous presence of different microbial populations within the same environment results in a highly competitive ecological niche, in which microbes compete for survival resources [26].

#### Mannanase activity

The increased mannanase activity observed in T2 and T3 can be attributed to the proliferation of mannanolytic bacteria derived from coconut-waste in the IMOS solution, which produce mannanase to hydrolyze mannan [27]. Fermentation periods of 10 and 15 days were identified as optimal for IMOS to achieve high mannanase activity. The mannanase activity recorded in coconut-waste–based IMOS, reaching 20.48 U/mL, was higher than that reported for A. sulphureus, which exhibited 16 U/mL of mannanase activity [28]. However, when the fermentation time was prolonged to 20 days, mannanase activity declined, likely because of mannanolytic bacteria entering the death phase due to the reduction of available nutrients in the IMOS medium.

### Tolerance to gastrointestinal conditions

#### Viability at gastrointestinal pH

In this study, the viability at pH 2.5 was higher than that previously reported, where probiotics from LAB isolated from ikan budu exhibited an average viability of 90% after 3 h and 75% after 6 h [29], while isolates obtained from okara survived at pH 2.5 for 2 h with 74.02% viability [30]. Based on the viability values at pH 2.5 for both 3 and 6 h, the LAB present in the coconut-waste fermentation medium can be categorized as robust and promising probiotic candidates. Viability at pH 2.5 reflects the acidic gastric environment; thus, high survival rates indicate that these LAB strains can withstand gastric conditions in poultry. Zommara *et al*. [31] reported that LAB with survival rates of up to 72% under acidic gastric conditions are suitable for the development of probiotics. Furthermore, Prima *et al*. [32] and Amenu *et al*. [33] emphasized that survival at low pH with viability ≥50% is a requirement for probiotic LAB.

A reduction in viability was observed at pH 2.5 from 3 to 6 h. This decrease is likely a result of the accumulation of acid stress surpassing the homeostatic capacity of LAB cells [34], resulting in the death of acid-sensitive strains, whereas acid-tolerant strains survived. Gastric acidity serves as the primary selective barrier for microbes before entering the intestine, and probiotic LAB must endure extreme gastrointestinal conditions, from the upper gastrointestinal tract to the intestine, where they subsequently colonize the intestinal surface [35]. In this study, the reduction in LAB viability at pH 2.5 from 3 to 6 h in IMOS was 15.26% in T3 and 8.67% in T4.

#### Bile salt tolerance

The high bile salt resistance observed in treatments T3 and T4 can be attributed to the extended fermentation period, which allows microbial succession dynamics, whereby strains more tolerant to fermentation conditions, including bile salts, become dominant. Consequently, the microbial community shifts toward a composition that is intrinsically more resistant to bile salts. Prolonged fermentation intensifies selective pressure and intramicrobial competition due to the accumulation of secondary metabolites that modify the microbial habitat. These altered environmental conditions confer a selective advantage to strains possessing adaptive mechanisms such as bile salt hydrolase activity, membrane composition modification, or stress-response gene expression, enabling only the most tolerant microbes to survive and dominate at later fermentation stages [36].

The bile salt resistance of LAB isolated from coconut-waste–based IMOS was higher than that of several LAB isolates from ikan budu, which exhibited maximum resistance of 59.56% and 54.56% at 0.3% and 0.5% bile salt concentrations, respectively [37]. LAB from coconut-waste–based IMOS demonstrated a bile salt resistance of 71.45%, indicating their strong potential as probiotic candidates. According to Marlida *et al*. [38], probiotic strains should exhibit a minimum survival rate of 20%–40%, with gastric acidity and bile salts being the primary barriers to survival.

#### Thermotolerance

During short fermentation (T1), the microbial community may not have adequately activated or synthesized protective components, such as EPS and stress-response enzymes, resulting in lower viability at elevated temperatures. By day 10 of fermentation (T2), the bacteria had begun synthesizing these protective components, thereby exhibiting greater tolerance at 42°C. However, prolonged fermentation (15 and 20 days) led to the accumulation of organic acids, a further decrease in pH, enhanced proteolytic activity, and the production of secondary metabolites that can damage cells [39]. These changes may shift the population toward strains with reduced thermotolerance.

### Hydrophobicity

The high hydrophobicity observed in T3 is associated with the elevated activities of mannanase and cellulase, which degrade mannan and fiber in coconut-waste into simple sugars. Bacteria subsequently utilize these sugars as substrates for polymerization into EPS through enzymatic processes. EPS production can alter the physicochemical properties of the cell surface, thereby enhancing the ability of LAB to adhere to the intestinal mucosa. EPS synthesis has been reported to modulate cell surface characteristics, often improving adhesion capacity and increasing cell surface hydrophobicity [40].

The hydrophobicity values obtained in the present study (T1 = 93.34%; T2 = 90.09%; T3 = 95.09%; T4 = 92.24%) were generally higher than those obtained in many previous reports, in which LAB isolates frequently exhibited hydrophobicity below 90%. For instance. Zhao *et al*. [41] reported a maximum hydrophobicity of 80.75% in LAB isolated from fermented substrates. Similarly, Shivani and Shatiyavelu [42] documented 85% hydro-phobicity in probiotics derived from *Murraya koenigii*, whereas Behbahani *et al*. [43] observed only 55.42% hydrophobicity in *Lacticaseibacillus paracasei* B31-2. These differences indicate that LAB from coconut-waste–derived IMOS exhibits relatively strong surface adhesion potential compared with many probiotic candidates recently reported in the literature.

### Antimicrobial activity against pathogenic bacteria

Supplementation with IMOS (P1–P4) reduced the pathogenic bacterial counts, although the degree of inhibition varied depending on the fermentation duration. This finding indicates that coconut-waste IMOS has inhibitory activity against pathogens such as *E. coli*, *S. aureus*, and *Salmonella*. A reduction in pathogen counts was observed in P1 (5-day fermentation), although the decrease was not statistically significant. This may be attributed to functional microbes in IMOS, particularly LAB, still being in the early logarithmic growth phase, resulting in suboptimal production of antimicrobial metabolites. This was evident from the relatively low LAB colony counts at the early fermentation stage (Table 1), resulting in limited antimicrobial activity. Similarly, Figueroa *et al*. [44] reported that the antimicrobial activity of LAB is strongly dependent on microbial composition and fermentation duration.

T2 treatment (10-day fermentation) exhibited a more pronounced inhibitory effect than T1. This is probably because by day 10, LAB populations had reached their optimal density, resulting in the accumulation of antimicrobial metabolites such as organic acids, hydrogen peroxide, and bacteriocins at higher concentrations. These compounds lower environmental pH and inhibit pathogen growth. The most pronounced inhibitory effect was observed in T3 (15-day fermentation), where *E. coli* failed to grow after 24 h. This suggests that the 15-day fermentation produced the most effective metabolite composition and microbial density to inhibit *E. coli*. Inhibition of *S. aureus* and *Salmonella* was also evident after 36 h, corresponding to the peak of the LAB exponential growth phase, when inhibitory metabolites accumulate at their maximum levels. LAB are known to produce organic acids (e.g., lactic and acetic acids), which reduce environmental pH, alongside antimicrobial metabolites such as bacteriocins, hydrogen peroxide, and secondary inhibitory compounds, particularly against pathogenic bacteria [45].

However, the inhibitory effect was reduced in T4 (20-day fermentation). This may be a result of decreased LAB viability caused by substrate depletion, cell autolysis, or shifts in the microbial community dominating the fermentation process. Such conditions may lower the concentrations of antimicrobial compounds, thereby diminishing the inhibitory capacity of IMOS compared with T2 and T3. Therefore, optimizing fermentation duration is essential to maximize the antimicrobial potential of IMOS [46]. Overall, these findings indicate that coconut-waste–derived IMOS effectively inhibits the growth of pathogenic bacteria, with the highest efficacy observed after fermentation for 10–15 days. This supports its potential as a natural probiotic candidate, consistent with one of the essential probiotic criteria: the ability to suppress pathogenic microbe colonization in the gastrointestinal tract.

### Short-chain fatty acids

The fatty acid profile obtained in this study confirmed that the microbial community in IMOS consistently produced acetate and butyrate during fermentation for 5–20 days. The relatively high concentration of acetate has major implications for pathogen inhibition, as acetate reduces environmental pH and disrupts the homeostasis of gram-negative bacteria such as *E. coli* and *Salmonella* [47]. Although butyrate concentrations were lower, its physiological significance is substantial. Butyrate serves as the primary energy source for colonocytes, strengthens the intestinal epithelial barrier, and exerts anti-inflammatory effects through *GPR109a* activation and *HDAC* inhibition [48].

The acetate concentration in this study, reaching 3.37 g/L, was higher than that reported in pineapple-based eco-enzymes (1.83 g/L) [49] but lower than that obtained from vegetable-based eco-enzymes, which reached 4.44 g/L [50]. The relatively low propionate content suggests that coconut-waste–based fermentation favors acetate and butyrate production pathways over propionate biosynthesis. Overall, the stable concentrations of SCFAs throughout fermentation reinforce the hypothesis that SCFAs are major contributors to the antimicrobial activity of IMOS, with acetate serving as the primary antimicrobial agent and butyrate functioning as an immune-modulatory metabolite.

### Practical implications and synbiotic relevance

The functional characteristics of IMOS identified in this study have important implications for poultry production, although in vivo validation has not yet been performed. High LAB viability under acidic, bile, and heat stress conditions indicates that IMOS-derived microbes are likely to survive passage through the gastrointestinal tract of poultry, where they may contribute to improved intestinal health. The detected mannanase activity suggests the release of MOS, a known prebiotic that enhances beneficial microbial colonization and reduces pathogen attachment in poultry. Furthermore, the production of SCFAs, especially acetate and butyrate, aligns with previous findings showing that these metabolites support epithelial integrity, modulate immune responses, and improve nutrient absorption. Collectively, these mechanisms suggest that IMOS has the potential to enhance poultry growth performance and feed efficiency; however, future in vivo trials are required to confirm this hypothesis.

The increased mannanase activity observed after 10–15 days of fermentation indicates efficient hydrolysis of mannans from coconut pulp into MOS. When combined with LAB populations in IMOS, these MOS molecules likely contribute to a synbiotic effect. MOS can selectively stimulate beneficial intestinal microbes, block pathogen adhesion to epithelial receptors, and enhance mucosal immunity, thereby complementing the probiotic functions of LAB. This mechanism supports the synbiotic role of IMOS-derived from coconut-waste.

The use of coconut water and pulp as substrates for IMOS production provides an environmentally sustainable approach, as these materials are often underutilized agricultural by-products that are readily available. Converting coconut-waste into functional synbiotic products not only reduces organic waste accumulation but also offers an affordable alternative to commercial probiotics, contributing to sustainable livestock production systems.

### Implications for poultry health and performance

Although this study was limited to in vitro evaluation, the observed characteristics of IMOS-derived from coconut-waste suggest relevant implications for poultry health. The high tolerance of LAB to acidic pH, bile salts, and elevated temperature indicates their potential to survive gastrointestinal transit and colonize the intestinal tract, where they may contribute to improved gut microbial balance [51, 52].

The antimicrobial activity against *E. coli*, *Salmonella* spp., and *S. aureus*, together with increased SCFA production, suggests a favorable intestinal environment that may support intestinal integrity and gut histological development, including increased villus height and improved villus-to-crypt ratio, as reported in poultry studies involving prebiotic and synbiotic supplementation.

In addition, SCFAs, such as acetate and butyrate, are known to play roles in immune modulation by enhancing epithelial barrier function and regulating inflammatory responses [53, 54], which may contribute to improved disease resistance in poultry. Collectively, these mechanisms are expected to translate into better nutrient utilization and growth performance when IMOS is applied in vivo, which warrants further validation through controlled poultry feeding trials.

### Limitations of the study and future research directions

This study was limited to the in vitro characterization of coconut waste–derived IMOS, and no in vivo trials were conducted in poultry. Therefore, the observed probiotic and synbiotic properties could not be directly linked to growth performance, gut histomorphology, immune responses, or feed efficiency under practical production conditions.

Additionally, LAB were evaluated at the community level without strain-level genetic identification, such as *16S rRNA* gene sequencing or whole-genome analysis. The specific contribution of individual microbial taxa and their safety traits could not be fully elucidated. Furthermore, shelf-life stability, long-term storage behavior, and comprehensive safety assessments, including hemolytic activity and antibiotic resistance profiling were not addressed in this study.

Future research should focus on controlled in vivo poultry trials to validate the functional effects of IMOS on gut health, immunity, and productivity. Molecular identification of dominant strains, safety evaluation, and shelf-life assessment are also required to support the practical application and commercialization of IMOS as a probiotic or synbiotic feed additive.

## CONCLUSION

This study provides a comprehensive *in vitro* evaluation of coconut waste–derived IMOS, demonstrating its strong probiotic and synbiotic potential for poultry applications. The results showed that fermentation duration critically influenced IMOS functionality, with 10–15 days identified as the optimal range. At this stage, IMOS exhibited the lowest pH, the highest LAB counts, elevated cellulase and mannanase activities, strong tolerance to acidic pH, bile salts, and elevated temperature, high cell surface hydrophobicity, pronounced antimicrobial activity against *E. coli*, *S. aureus*, and *Salmonella* spp., and stable production of SCFAs, particularly acetate and butyrate.

From a practical perspective, these findings indicate that IMOS can serve as a low-cost, locally available alternative to AGPs in poultry production. High LAB viability under simulated gastrointestinal conditions suggests a strong likelihood of survival during gastrointestinal transit, while antimicrobial activity and SCFA production support pathogen suppression and intestinal health. The observed mannanase activity indicates MOS release, supporting a synbiotic mechanism that may enhance beneficial microbial colonization and reduce pathogen adhesion in the poultry gut.

A key strength of this study lies in its integrated approach, simultaneously linking microbial growth dynamics, enzymatic functionality, gastrointestinal stress tolerance, antimicrobial effects, and SCFA production within a single fermentation system. This holistic evaluation provides a clearer understanding of IMOS functionality compared with studies focusing on isolated probiotic attributes.

In conclusion, coconut waste–derived IMOS exhibits robust probiotic and synbiotic characteristics and represents a sustainable strategy for improving poultry gut health and reducing reliance on AGPs. While the findings strongly support its functional potential, further in vivo validation is required to confirm its effects on growth performance, gut morphology, immune responses, and feed efficiency under practical poultry production conditions.

## DATA AVAILABILITY

All the generated data are included in the manuscript.

## AUTHORS’ CONTRIBUTIONS

HDT: Conceptualization, study design, supervision, data analysis and interpretation, manuscript drafting and revision. MAA: Methodology, data analysis, and manuscript revision. YM: Conceptualization, supervision, data interpretation, and manuscript revision. IEP and TM: Laboratory experiments, data collection, and preliminary analysis. GY: Data curation and manuscript drafting. RH: Statistical analysis and data validation. WDA: SCFA analysis and interpretation. RP: Formal data analysis, Data interpretation, critical revision of the manuscript, and final approval. All authors approved the final manuscript and agree to be accountable for all aspects of the work
